# Fourteen Years of Archaeological and Heritage Research in the Iringa Region, Tanzania

**DOI:** 10.1007/s10437-020-09383-w

**Published:** 2020-05-20

**Authors:** J. M. Miller, J. J. Werner, K. M. Biittner, P. R. Willoughby

**Affiliations:** 1 Department of Anthropology, University of Alberta, Edmonton, AB Canada; 2 Department of Archaeology, Max Planck Institute for the Science of Human History, Jena, Germany; 3 Department of Anthropology, Economics and Political Science, MacEwan University, Edmonton, AB Canada

**Keywords:** Stone Age, Mkwawa, Isimila, Lithics, Tanzania, Iringa, Acheulean, Iron Age

## Abstract

The Iringa Region is famous among archaeologists for the Acheulean site of Isimila, and among historians as the stronghold where Chief Mkwawa led the Hehe resistance against German colonial forces. However, our research reveals that Iringa has a rich archaeological record that spans the period from the Stone Age into the recent past. This article summarizes the results of 14 years of research by our team, the Iringa Region Archaeological Project (IRAP). Since 2006, IRAP members have recorded 67 sites, and this only scratches the surface of the archaeological potential in the area. These sites, some of which were recorded in conjunction with local participants, have archaeological component characteristic of the Early, Middle, and Later Stone Age, the Iron Age, and the recent past. We consider the archaeological and historical value of Iringa to be high and hope that this work inspires future research, tourism, and conservation efforts in the area.

## Résumé

La région d’Iringa en Tanzanie est. reconnue par les archéologues pour le site acheuléen d’Isimila et parmi les historiens comme le fief où le chef Mkwawa a dirigé la résistance du Hehe contre les forces coloniales allemandes. Cependant, nos recherches révèlent qu’Iringa possède de riches archives archéologiques allant de l’âge de pierre au passé récent. Cet article résume les résultats de quatorze années de recherche de notre équipe, le projet archéologique de la région d’Iringa (PARI). Depuis 2006, les membres du PARI ont enregistré 67 sites, ce qui ne fait qu’effleurer la surface du potentiel archéologique de la région. Les sites identifiés présentent des éléments archéologiques caractéristiques de l’âge de pierre précoce, moyen et postérieur, de l’âge du fer, et du passé historique. En outre, un certain nombre de sites culturels importants ont été enregistrés en collaboration avec des participants locaux. Nous considérons que la valeur archéologique et historique d’Iringa est. élevée et espérons que ces travaux inspireront la recherche, le tourisme et la conservation dans cette région importante.

## Introduction

**Fig. 1:**
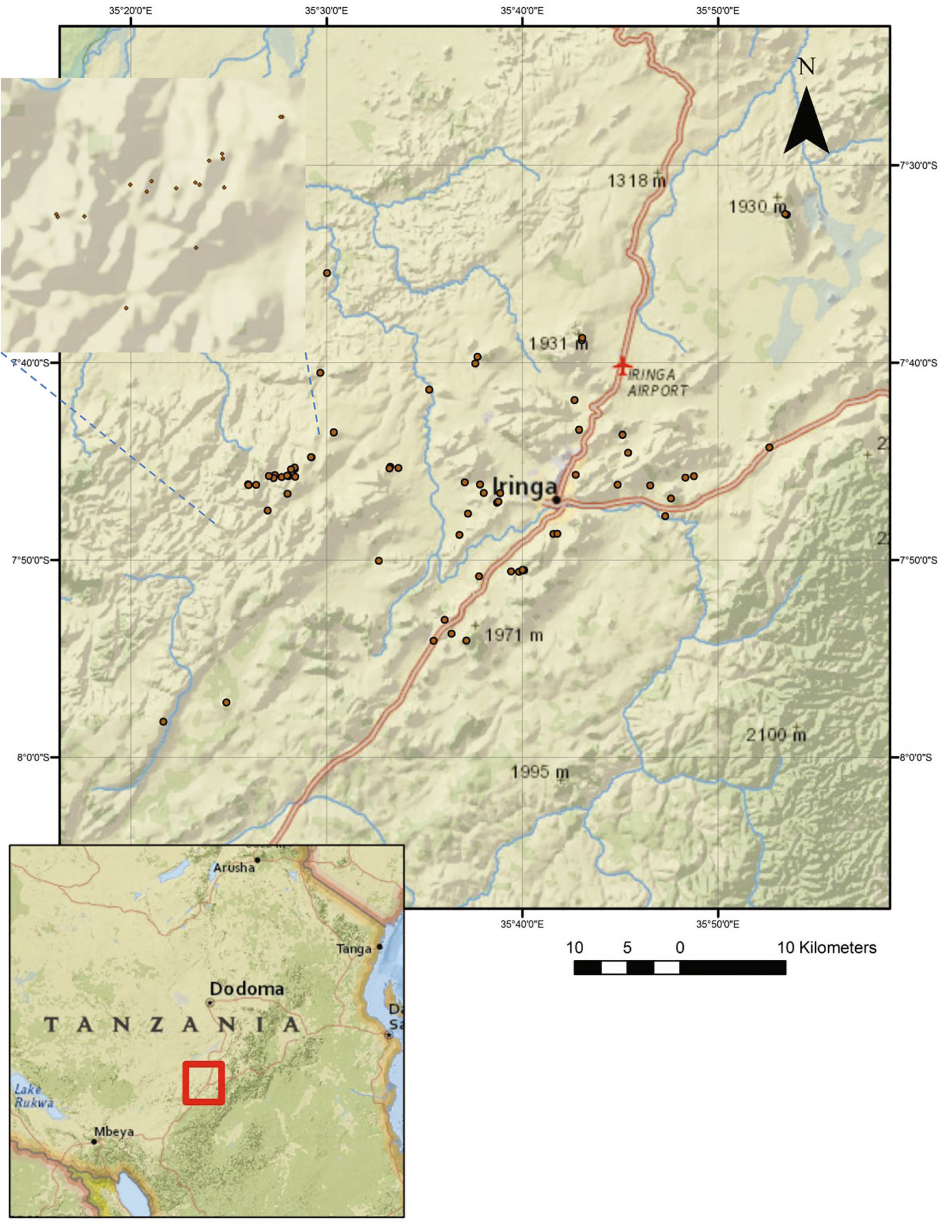
Sites identified in the Iringa Region

While archaeological research in Tanzania has traditionally focused on the northern and coastal regions of the country, our project has spent the last 14 years documenting the rich record of the country’s southern highlands. We have been investigating the Iringa Region since 2005 when Dr. Pamela Willoughby was first shown several rockshelters that appeared to have remarkable archaeological potential. Since that time, we have recorded 67 archaeological sites in a roughly 3600 km^2^ area. These sites demonstrate the intensive occupation of Iringa from the earliest phases of human evolution through the present (Fig. 1).

In the 1980s, geneticists and paleontologists observed that the first anatomically modern humans were African, and were associated with MSA artifacts (Cann et al. 1987; Grine 2016; Stringer and Andrews 1988). In eastern Africa, this period was only known from a few sites located primarily in the Eastern or Gregory Rift Valley in Kenya, Ethiopia, and northern Tanzania. In order to expand this geographic range, Willoughby began an investigation of southern Tanzania, searching for new MSA localities. In the late 1980s and early 1990s, Willoughby surveyed the Mbeya Region, part of the Western or Albertine Rift Valley, which bordered what was then the Iringa Region. She documented open-air Stone Age sites, many of which were located along terraces of the Songwe River, which flows into Lake Rukwa (Willoughby 2001; Willoughby and Sipe 2002). Unfortunately, in addition to taphonomic disturbance, these archaeological sites lacked organic preservation. The only places with good preservation and in situ material seemed to be rockshelters.

Seeking MSA rockshelter sites, Willoughby moved into the neighboring Iringa Region. The landscape of Iringa is speckled with granite boulders, which erode out of the hillsides and form natural shelters. These features make Iringa attractive for archaeological research. Her initial visit to several Iringa sites in 2005 confirmed this potential, and the Iringa Region Archaeological Project (IRAP) was born. This article summarizes the results of archaeological work carried out by members of IRAP, since that first visit in 2005. Here we list and describe sites from the Early Stone Age (ESA), Middle Stone Age (MSA), Later Stone Age (LSA), Iron Age (IA), and recent periods. While Willoughby’s initial goal was to document MSA rockshelter sites, it quickly became clear that this is only part of what Iringa has to offer. The goal, therefore, evolved to highlight the full spectrum of the archaeological significance of Iringa. We hope this article will encourage further research and conservation efforts and tourism development in this important area.

## Geography—Past and Present

The Iringa Region is located in the southern highlands of Tanzania, at an elevation of 1550 m (or 5090 ft) above sea level. Although it is classified as a hot, arid steppe biome in the worldwide Köppen-Geiger climate classification system (Beck et al. 2018), the high elevation reduces temperatures considerably, and winter nights can drop to near freezing. The mean annual air temperature is ≥ 18 °C, with a mean annual precipitation of 750–1000 mm (almost exclusively during the rainy season, December–March). The flora of Iringa is characterized by moist savanna and dry montane vegetation, particularly evident on hill slopes where modern cultivation is limited. While specific palaeoenvironmental data for the region is currently lacking, analysis of nearby areas shows a relatively mild and stable climate over the last 75,000 years (Cohen et al. 2007; Scholz et al. 2007). It may be that the climate stability attracted early hominin groups to Iringa, resulting in its continuous long-term occupation.

Some of the most striking features of the Iringa landscape are granitic inselbergs and steep ravines, both of which hold useful archaeological information. The inselbergs are of the bornhardt variety, broken down into castle kopjes (Buckle 1977). These result in rocky outcrops (called *mapango* in Swahili) that provided natural shelters and are commonly associated with cultural materials. The erosional gullies (*makorongo* in Swahili) expose ancient archaeological materials that would otherwise lie deep underground. These ravines are created when ephemeral streams flow down steep-sided hills during the wet season. As such, they are typically located along the bases of the Udzungwa Mountains and foothills in Iringa.

**Fig. 2:**
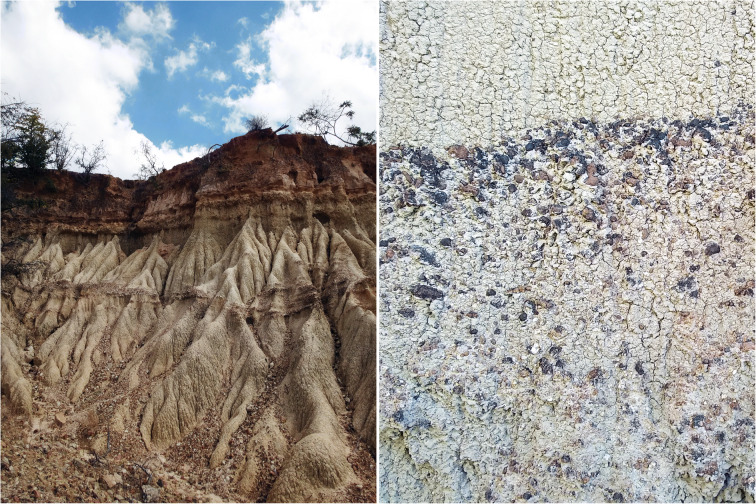
Lag deposits (horizontal bands), often observed in erosional gullies in Iringa

During the 2016 and 2018 field seasons, we observed high concentrations of cultural materials associated with or directly embedded in heavily varnished gravel layers exposed by erosion. These distinct layers are lag deposits (also known as colluvial lag or desert pavements), which form through natural processes when wind and water remove small sedimentary grains (clay, silt, sand, gravel) from the surface (Fig. 2). Over time, this process deflates the depositional surface leaving the larger, heavily weathered rocks, including artifacts, behind. As the deposit stabilizes, the larger particles form a hardened “pavement” cap on the deposit and protect underlying layers from further erosion. Lag deposits do not represent discrete depositional times or events. Instead, they are often composed of distinct deposits that have collapsed into a single layer.

The availability of raw materials for lithic tools was a paramount consideration during the Stone Age, and the Iringa Region provides a variety of these stone materials. White quartzite of moderate to good quality is abundant and can be found throughout the region in nodules of varying sizes. Other raw materials include a series of other metamorphic rocks, quartz, and chert (cryptocrystalline silica). It appears that although most raw materials are only of moderate quality, their quality and relative abundance made them a sufficient resource to satisfy the demand for tools from the ESA onwards. For a comprehensive review of the geology of Iringa, see Biittner (2011), with supplementary analyses of the lithic raw material economy in Alexander (2010), and Werner and Willoughby (2017).

## Survey Methods

IRAP made use of two non-probabilistic approaches to identify new sites in the region. First, we engaged with the local people who shared their knowledge of the landscape with us. Sites identified in this manner are often primarily of modern, historical, and cultural significance, although some (such as Mlambalasi Rockshelter) have also yielded deep-time archaeological components. Second, we employed remote sensing technologies, specifically satellite imagery from Google Earth. This approach has allowed our team to identify natural exposures and unique topographic features beforehand, conserving the valuable and limited time in the field. These survey strategies tend to overemphasize certain areas, resulting in clusters of sites that do not necessarily reflect past land-use patterns. Nevertheless, our results demonstrate that different human populations occupied the Iringa landscape from the ESA through modern times.

The sites identified in Tanzania are assigned a unique designation with the Standardized African Site Enumeration System (SASES) (Nelson 1971) and reported along with GPS coordinates and supplemental information to the Tanzanian Division of Antiquities, Ministry of Natural Resources and Tourism. In addition to this standardized naming system, IRAP documented the local name of each site whenever possible, usually referring to the names of the area, or the closest road. Convention suggests that sites be numbered based on the order of discovery. However, it can be a challenge to access information about prior archaeological work in Iringa. Many of the previous works are documented in the form of government reports. And, it is not always possible to know whether a site has been already studied, or which SASES numbers were used. In areas where we suspected previous work had taken place, site numbers were assigned, starting at 100 rather than 1, thereby avoiding duplicate designations.

While conducting fieldwork, IRAP disseminated knowledge about archaeology to local communities through a variety of means. We formally shared our findings in community meetings, museum collaborations, informational posters, and brochures. We also informally talked to any interested residents about our project, in an attempt to promote awareness for the rich archaeological history of Iringa. In December 2018, members of our team participated in the *Utalii Karibu Kusini* Fair and collaborated with our colleagues in the heritage and related industries to promote the visibility of the Iringa Region and its cultural heritage. In some cases, we encounter modern activities such as quarrying, construction, or cultivation that have exposed artifact-bearing layers. Several scholars have discussed the impacts of these contemporary economic activities on cultural heritage and archaeological sites in Tanzania (e.g., Bushozi 2014; Masele 2012).

## Culture History of Iringa

Our work has recorded 65 new sites, bringing the total of known sites in the Iringa Region to 67 (Table 1). The list includes archaeological sites (ESA, MSA, LSA, and IA), as well as culturally significant sites from the recent period. The majority of these sites are located in rockshelters (*n* = 27) and erosional gullies (*n* = 20). Nearly every one of these landforms we assessed had archaeological material, so it is certain that further exploration will yield additional sites. In this section, we describe the archaeological sequence in Iringa, moving from the earliest periods to the most recent, and summarize the characteristic finds.

**Table 1:** List of sites identified in the Iringa Region

SASES #	Common name or description	GPS coordinates	Site type	Cultural components			
				ESA	MSA	LSA	IA
HwJf-1	Mkwawa’s Monument	7° 35′ 34.200″ S, 35° 30′ 07.000″ E	Cultural				
HwJf-2	Mlambalasi Rockshelter	7° 35′ 27.773″ S, 35° 30′ 1.696″ E			y	y	y
HwJf-3	Nzihi 1	7° 40′ 31.020″ S, 35° 29′ 40.980″ E	Open-air		y	y	y
HwJf-4	Nzihi 2	7° 44′ 47.640″ S, 35° 29′ 14.160″ E	Shelter				y
HwJf-5	Nzihi 3	7° 44′ 47.400″ S, 35° 29′ 12.600″ E	Shelter				y
HwJg-1	Kidamali Hill	7° 43′ 32.300″ S, 35° 30′ 21.900″ E	Shelter				y
HwJg-2	Mgongo/Ndalogwa	7° 41′ 54.180″ S, 35° 42′ 40.020″ E	Gulley	y			
HwJg-100	Kihesakilolo	7° 43′ 24.660″ S, 35° 42′ 53.940″ E	Shelter		y	y	y
HwJg-101	Kikongoma	7° 41′ 22.740″ S, 35° 35′ 14.820″ E	Cultural				
HwJg-102	Mapanda Rockshelter 1	7° 38′ 44.459″ S, 35° 43′ 3.644″ E	Shelter				y
HwJg-103	Mapanda Rockshelter 2	7° 38′ 51.734″ S, 35° 43′ 2.276″ E	Shelter				y
HwJg-104	Mlangali Hill	7° 40′ 3.050″ S, 35° 37′ 35.652″ E	Gulley		y	y	y
HwJg-105	Mgera Furnace Site	7° 39′ 42.131″ S, 35° 37′ 42.784″ E	Open-air		y	y	y
HwJh-1	Kigonzile Korongo 1	7° 44′ 33.810″ S, 35° 45′ 24.264″ E	Gulley	y	y		y
HwJh-2	Kigonzile Korongo 2	7° 43′ 39.695″ S, 35° 45′ 7.312″ E	Gulley		y	y	
HwJh-3	Mafinga Rockshelter 1	7° 32′ 30.060″ S, 35° 53′ 30.720″ E	Cultural/shelter				y
HwJh-4	Mafinga Rockshelter 2	7° 32′ 27.600″ S, 35° 53′ 25.500″ E	Shelter				y
HwJh-5	Lugalo Monument	7° 44′ 18.060″ S, 35° 52′ 36.960″ E	Cultural				
HxJe-1	Malunde/Tungamalenga	7° 51′ 54.000″ S, 35° 6′ 32.940″ E	Open-air/cultural				y
HxJf-1	Magubike Rockshelter	7° 45′ 23.158″ S, 35° 28′ 22.804″ E	Shelter		y	y	y
HxJf-2	Magubike Rockshelter 2	7° 47′ 30.000″ S, 35° 27′ 0.000″ E	Shelter			y	y
HxJf-3	Magubike Field	7° 45′ 47.400″ S, 35° 28′ 23.940″ E	Open-air		y		y
HxJf-4	Magubike Rockshelter 3	7° 47′ 30.050″ S, 35° 27′ 0.050″ E	Shelter			y	y
HxJf-5	Magubike Rockshelter 4	7° 45′ 18.960″ S, 35° 28′ 22.320″ E	Shelter			y	y
HxJf-6	Kitwilu Rockshelter	7° 45′ 42.060″ S, 35° 27′ 21.780″ E	Shelter			y	y
HxJf-7	Magubike iron furnaces 1	7° 45′ 48.180″ S, 35° 27′ 42.720″ E	Open-air				y
HxJf-8	Magubike Rockshelter 5	7° 45′ 51.060″ S, 35° 27′ 17.940″ E	Shelter			y	y
HxJf-9	Magubike Rockshelter 6	7° 45′ 44.940″ S, 35° 27′ 3.240″ E	Shelter			y	y
HxJf-10	Magubike gully	7° 45′ 43.560″ S, 35° 27′ 59.160″ E	Gulley		y		
HxJf-11	Magubike iron furnaces 2	7° 45′ 24.540″ S, 35° 28′ 11.400″ E	Open-air				y
HxJf-12	Rock art	7° 46′ 10.380″ S, 35° 25′ 59.700″ E	Shelter			y	y
HxJf-13	Iron slag accumulation	7° 46′ 11.940″ S, 35° 26′ 23.940″ E	Open-air				y
HxJf-14	Kwilamba/Kiponzelo	7° 58′ 11.760″ S, 35° 21′ 39.900″ E	Cultural			y	y
HxJf-15	Ilyana/Luteme	7° 57′ 13.320″ S, 35° 24′ 53.340″ E	Shelter			y	y
HxJf-16	Songumbele Rockshelter	7° 46′ 39.061″ S, 35° 28′ 0.167″ E	Shelter			y	y
HxJf-17	Kwayowe Rockshelter	7° 45′ 45.414″ S, 35° 28′ 2.845″ E	Shelter		y	y	
HxJf-18	Lutona Rockshelter	7° 46′ 12.700″ S, 35° 26′ 1.200″ E	Shelter				y
HxJg-6	Isimila	7° 53′ 44.130″ S, 35° 36′ 23.110″ E	Gulley	y			
HxJg-100	Lukingi Hill Rockshelter	7° 54′ 5.372″ S, 35° 37′ 8.517″ E	Shelter			y	
HxJg-101	Iringa Girls Secondary School	7° 45′ 41.040″ S, 35° 42′ 43.500″ E	Shelter		y	y	
HxJg-102	Lupuli at Kalenga	7° 48′ 44.280″ S, 35° 36′ 47.520″ E	Open-air	N/A	N/A	N/A	N/A
HxJg-103	Kigwabimbi	7° 47′ 39.420″ S, 35° 37′ 13.200″ E	Gulley		y	y	y
HxJg-104	Kibebe	7° 50′ 2.340″ S, 35° 32′ 40.320″ E	Open-air	N/A	N/A	N/A	N/A
HxJg-105	Isimila 2	7° 54′ 5.940″ S, 35° 35′ 27.840″ E	Open-air	y	y		
HxJg-106	Mseke	7° 50′ 49.800″ S, 35° 37′ 47.340″ E	Shelter			y	y
HxJg-107	Mangalali Hill	7° 45′ 20.182″ S, 35° 33′ 40.288″ E	Open-air		y	y	y
HxJg-109	Mangalali iron furnaces	7° 45′ 21.420″ S, 35° 33′ 13.200″ E	Open-air				y
HxJg-112	Kitasengwa Korongo 1	35° 37′ 50.096″ E, 7° 46′ 10.786″ S	Gulley	y	y	y	
HxJg-113	Kitasengwa Korongo 2	7° 46′ 4.026″ S, 35° 37′ 3.882″ E	Gulley			y	
HxJg-114	Kitisengwa Korongo 3	7° 46′ 36.900″ S, 35° 38′ 52.260″ E	Gulley	y	y		y
HxJg-115	Njiapanda Korongo	7° 47′ 3.120″ S, 35° 38′ 46.740″ E	Gulley	y	y	y	y
HxJg-116	Kitasengwa Korongo 4	7° 47′ 5.977″ S, 35° 38′ 42.670″ E	Gulley	y	y	y	y
HxJg-117	Kitasengwa Korongo 5	7° 46′ 36.368″ S, 35° 38′ 1.968″ E	Gulley	y	y	y	y
HxJg-118	Mafifi Rockshelter	7° 50′ 32.183″ S, 35° 40′ 0.883″ E	Shelter				y
HxJg-119	Kitwiru iron furnaces	7° 50′ 31.135″ S, 35° 40′ 5.387″ E	Open-air				y
HxJg-120	Kitwiru Rockshelter	7° 50′ 29.929″ S, 35° 40′ 0.570″ E	Shelter				y
HxJg-121	Kitwiru Korongo	7° 50′ 34.595″ S, 35° 39′ 25.430″ E	Gulley	y	y	y	y
HxJg-122	Kisiwani Korongo 1	7° 48′ 41.339″ S, 35° 41′ 35.088″ E	Gulley	y	y	y	y
HxJg-123	Kisiwani Korongo 2	7° 48′ 40.684″ S, 35° 41′ 47.440″ E	Gulley	y	y	y	y
HxJg-124	Mangalali surface scatter	7° 45′ 16.790″ S, 35° 33′15.156″ E	Open-air		y		
HxJg-125	Kitwiru Rockart Rockshelter	7° 50′ 35.905″ S, 35° 39′ 49.990″ E	Shelter			y	y
HxJh-1	Ulonge Korongo	7° 46′ 53.308″ S, 35° 47′ 35.515″ E	Gulley				y
HxJh-2	Itabale Kigungawe Korongo	7° 46′ 13.560″ S, 35° 46′ 32.197″ E	Gulley		y	y	y
HxJh-3	Matunguu Korongo	7° 45′ 49.920″ S, 35° 48′ 19.680″ E	Gulley	y	y	y	y
HxJh-4	Ilange	7° 47′ 47.515″ S, 35° 47′ 18.103″ E	Open-air		y	y	y
HxJh-5	Kihanga Korongo	7° 45′ 45.364″ S, 35° 48′ 45.695″ E	Gulley	y	y	y	y
HxJj-1	Magombelema Rockshelter	7° 47′ 11.460″ S, 36° 23′ 33.600″ E	Cultural/shelter				y

### Early Stone Age

The ESA is present in Iringa though, to date, it has only been identified from natural exposures and not from excavated contexts. The ESA is best known in our study area from the site of Isimila, documented in the early 1950s (Howell 1961; Howell et al. 1962). The site was recently reexamined, using an uncrewed aerial vehicle to document erosion and topographic changes as well as archaeological features (Bergstrom et al. 2019). Isimila is a large erosional gully, famous for the hundreds of Acheulean hand axes, picks, and cleavers eroding out of the ravine walls. While it is best known for its Acheulean artifacts, the site also features MSA and LSA components. It was one of the first places to be designated as a National Monument by the government of the Republic of Tanzania, which means it receives protection under the Antiquities Act number 10 of 1964. As argued by Bergstrom et al. (2019), there is great potential for highly rewarding future archaeological excavations and for research that addresses the impact of erosion and other site formation processes in the area.

**Fig. 3:**
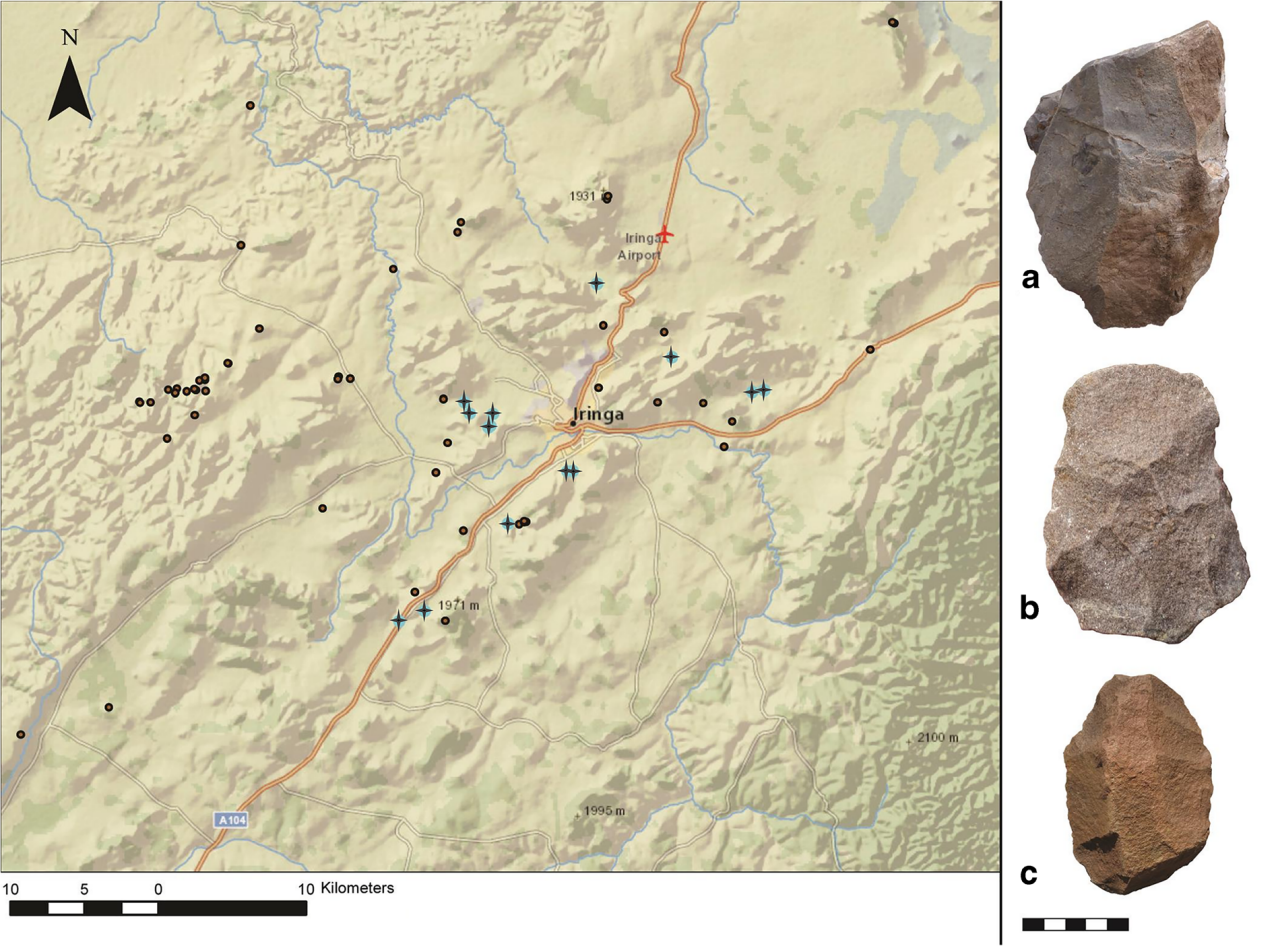
Distribution of ESA sites (shown with plus (+) symbols), and characteristic finds: **a** Large cutting tool or core from Kitisengwa Korongo 3; **b** Biface from Kitisengwa Korongo 3; **c** Biface from Kihanga Korongo (artifact scale in cm)

Including the world-famous Isimila, and the lesser-known site of Mgongo (Omi 1988), our research brings the total number of sites with ESA components in Iringa to fourteen (Fig. 3). These are dominated by Acheulean technology, primarily hand axes. While we did observe some core tools and choppers on the surface, these are less useful as a diagnostic element, and the lack of context of surface finds makes it unclear whether these artifacts are from an Oldowan or Acheulean component. Further research is required to confirm whether there is an Oldowan component in the Iringa Region and to chronometrically date the onset of the ESA.

### Middle Stone Age

**Fig. 4:**
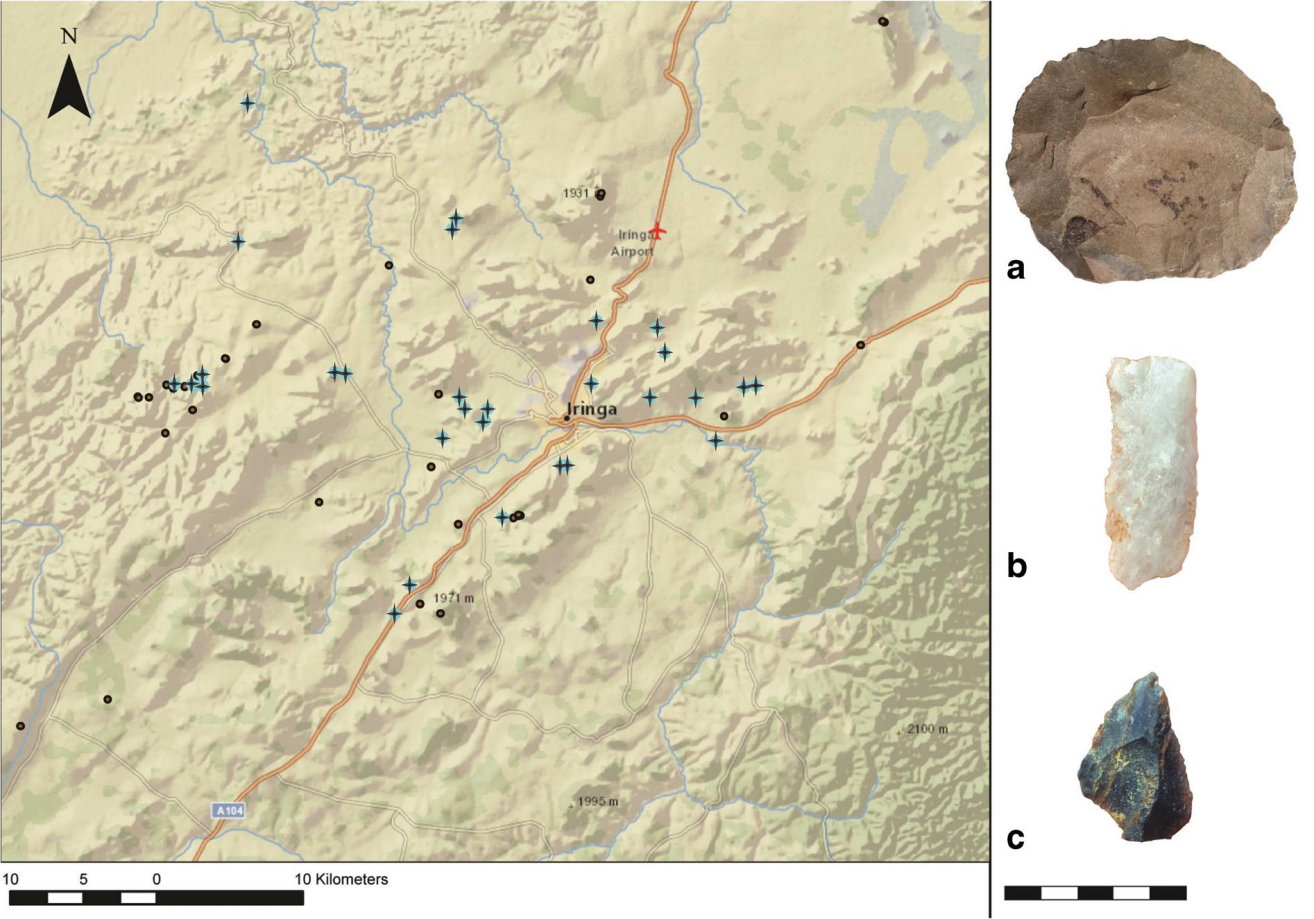
Distribution of MSA sites (shown with plus (+) symbols), and characteristic finds: **a** Levallois core from Kitisengwa Korongo 3; **b** Levallois blade from Kisiwani Korongo 1; **c** Levallois point from Kiponzile Korongo (artifact scale in cm)

The MSA is well-represented in the Iringa Region, and here we report 28 sites with MSA components (Fig. 4). A little more than half (*n* = 15) of these locations are erosional gullies with surface finds that are typologically diagnostic of the MSA. Typical artifacts include Levallois points/blades/cores, large scrapers, and occasionally backed tools, though it is difficult to distinguish late Acheulean from early MSA, as these two periods overlap in time and artifact types. Faunal remains are found in excavated contexts beginning in the MSA (Collins 2010; Collins and Willoughby 2010, and Masele 2017). As is the case for the ESA, there are no radiometric dates available for the onset of the MSA in Iringa, though early finds from other parts of eastern Africa suggest it could be as ancient as 320,000 years ago (Brooks et al. 2018).

Magubike Rockshelter contains the only excavated MSA context in Iringa, and has therefore received the most attention from IRAP (e.g., Miller and Willoughby 2014; Werner and Willoughby 2017; Willoughby et al. 2018). The Magubike MSA lithics show some reliance on radial or Levallois methods of blank production. However, the majority of cores are produced with the simple bipolar reduction method to create several scalar pieces (*outils écaillés*). As is typical with MSA assemblages, there are few retouched tools. The most numerous tool types are points and scrapers, with occasional large (> 5 cm) backed pieces. The raw materials are dominated by quartz, quartzite, and other metamorphic rocks but with the occasional presence of higher quality, curated materials such as chert (Bushozi 2011; Werner and Willoughby 2018).

The Iringa Region’s MSA deposits also feature the oldest direct dated ostrich eggshell (OES) beads in the world. Excavations at Magubike in 2012 recovered 39 OES beads/preforms, some of which were directly radiocarbon-dated to 31,810 ± 180 BP (OxA-27,627), 47,750 ± 750 BP (OxA-27,626), and > 50,100 BP (OxA-27,628) (Miller and Willoughby 2014, p. 120). This is the first time that OES beads have been found in association with MSA deposits, indicating that the OES bead-making tradition has ancient roots in Iringa.

### Later Stone Age

**Fig. 5:**
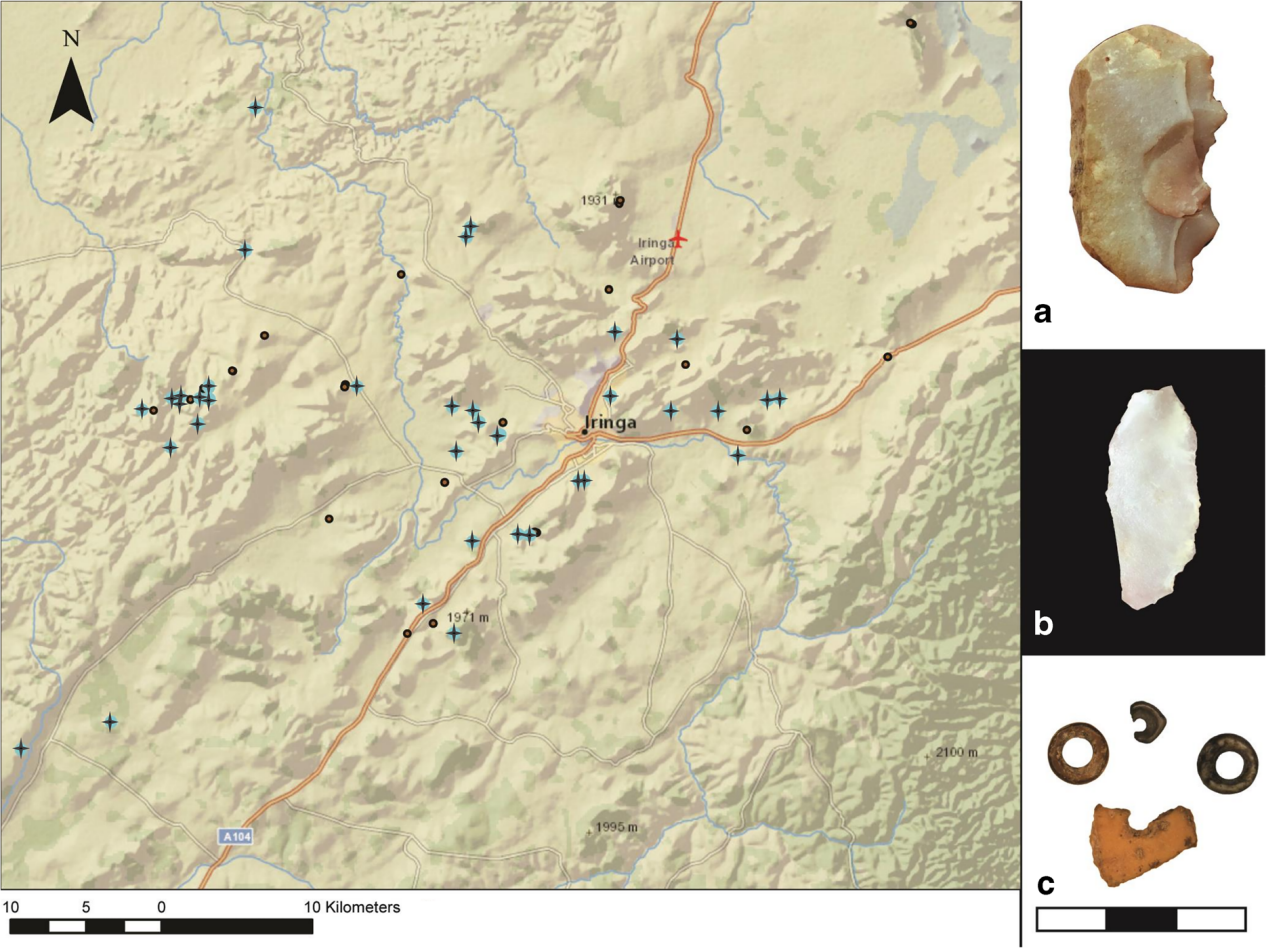
Distribution of LSA sites (shown with plus (+) symbols), and characteristic finds: **a** Backed geometric tool from Mangalali Hill; **b** Microblade from Kitisengwa Korongo 5; **c** OES beads and preforms from Magubike and Mlambalasi Rockshelters (artifact scale in cm)

Our understanding of the LSA in Iringa is still developing, and much of what we know is based on an analysis of excavated material from Magubike and Mlambalasi Rockshelters (Biittner et al. 2017). In the absence of clear stratigraphic layers or radiometric dates, lithic typology is often employed by IRAP to date and describe the cultural sequence in Iringa. The LSA lithics tend to include small (< 5 cm) geometric pieces, typically produced from white quartzite. On the basis of these criteria, we identified an LSA component at 36 sites (Fig. 5).

Broadly, there was technological continuity within the LSA in Iringa, but the size of blanks and finished tools gradually decreased over time. For example, at both Mlambalasi and Magubike, a microlithic LSA assemblage overlies a macrolithic one. At Mlambalasi, the microlithic assemblage dates to the Holocene, while the macrolithic level is dated to the Late Pleistocene. Low to moderate quality quartzite and quartz (including crystalline quartz) dominate the LSA lithic assemblages, and there is an overall reduction in the diversity of rock types used for lithic manufacture during the LSA. This decline in the use of raw materials such as cherts and metamorphics is possibly due to a focus on producing small tools that can be hafted, which does not require particularly fine-grained or high-quality raw materials. Bipolar reduction is still the dominant choice of blank production in the LSA, a practice that began in the MSA and continued through to IA and historical periods. A well-developed blade industry, which requires good quality raw material, does not seem to have existed at any time in the area around Iringa. We have recovered no more than a handful of potential blades and blade cores in the last decade of work, and we suspect that the few LSA blades that we do find may be unintended byproducts of other processes. Larger blades would likely be indicative of an MSA context.

The LSA at Mlambalasi is notable for having yielded the nearly complete remains of a primary human internment (Biittner et al. 2017; Sawchuk 2012; Sawchuk and Willoughby 2015). The remains, excavated in 2006 and 2010, show a high degree of taphonomic alteration from compaction, trampling, and termite activity. Despite these post-depositional changes, the individual can be identified as a middle-aged adult (35–50 years old) of small stature (Sawchuk 2012). Direct dating of the remains was not possible due to degradation of the bone collagen; however, OES beads from the burial context have been dated to 14,115 ± 55 BP (OxA-27,621), 14,275 ± 55 BP (OxA-27,623), and 16,690 ± 65 BP (OxA-27,624), suggesting this individual is from the terminal Pleistocene.

### Iron Age

**Fig. 6:**
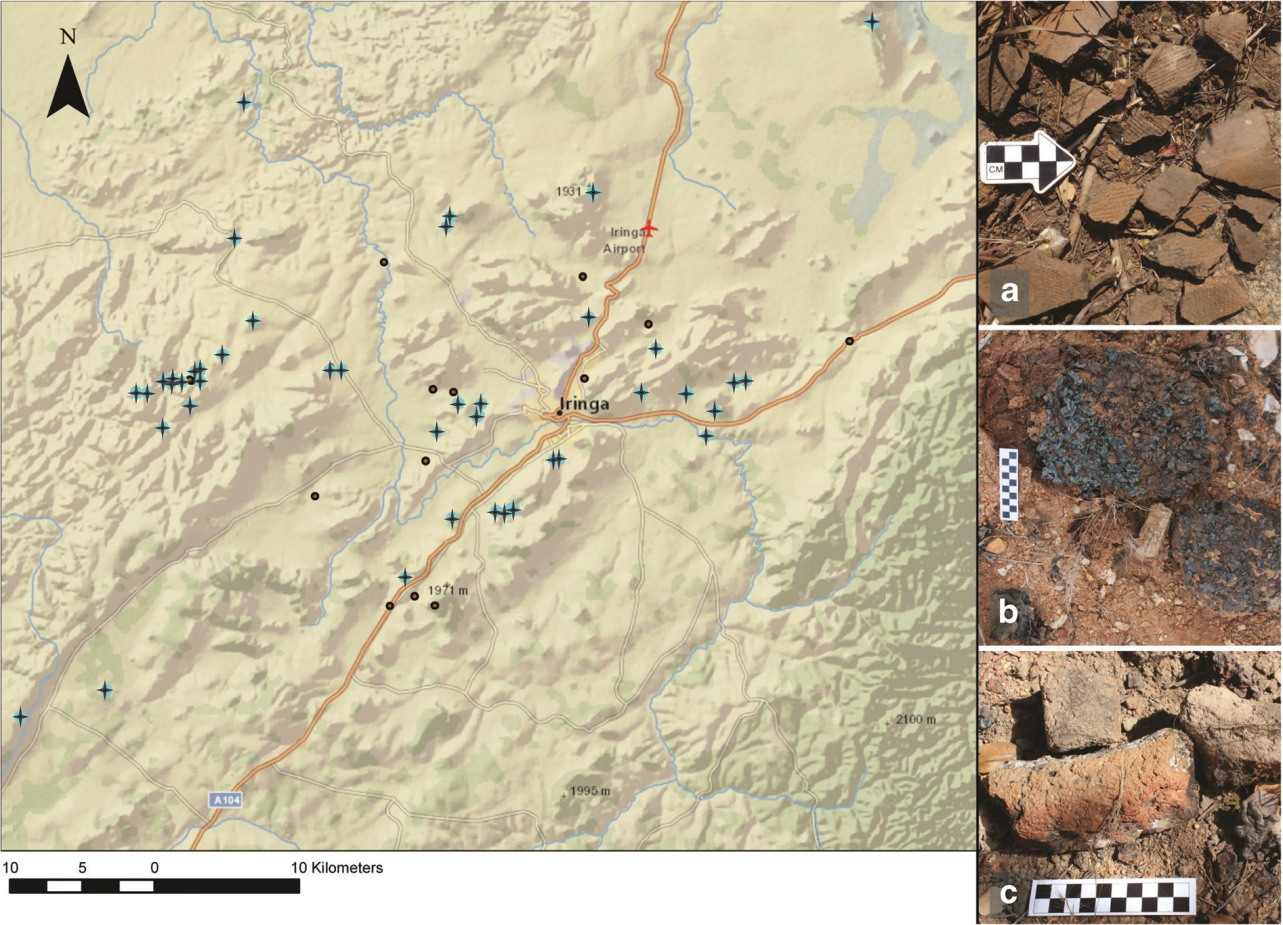
Distribution of IA sites (shown with plus (+) symbols), and characteristic finds: **a** Potsherd scatter from Ilange; **b** Iron slag from Ulonge Korongo; **c** Remnants of iron furnace from Kitwiru Korongo (artifact scales in cm)

Ironworking is prevalent in Iringa, and we have identified 51 sites with Iron Age components (Fig. 6). The Iron Age began around 2500 BP in Tanzania with the smelting of iron ore. Early Iron Age furnaces in Tanzania were constructed from bricks or clay rolls and were generally bowl-shaped, although tall shaft-like structures developed later (Lyaya and Mapunda 2016). At the base of the furnace are ports for tuyeres (the blowpipes which force air into the furnace to aid combustion). The remains of these iron furnaces often include fragments of clay walls, tuyeres, iron, and slag. The Mgera and Mangalali furnace sites, for example, feature extensive concentrations of debris from ironworking activities. Both sites have the in situ remains of several furnaces, which are evident as discrete scatters of furnace fragments, tuyere fragments, iron slag, and iron pellets.

Sites were categorized as Iron Age based on the presence of iron processing materials or ceramics. Ceramic artifacts were a common find in many of the places that were surveyed, and are often attributed to the Iron Age. In total, potsherds were documented in 44 sites. However, as ceramic vessels are still being made and used today, especially in the rural areas around Iringa town, there is no guarantee that these finds are ancient. This is especially true for sites near modern settlements. Potsherds from excavations at Mlambalasi Rockshelter are coarse and porous in texture and relatively thick and uneven in cross section; these characteristics, along with evidence of partial oxidation or burning, suggest the vessels were created using a pinching, or drawing technique and then fired directly in a hearth (Biittner et al. 2017).

No comprehensive study of ceramic vessel decoration has been conducted for Iringa. A variety of incised, stamped, punctated, rouletted, and applique designs have been observed on ceramics collected from the surface and in the excavated contexts throughout the region. These are similar to what has been reported elsewhere for early ironworking pottery traditions in southern coastal Tanzania (Kwekason 2013). A chronology of the ceramic decoration motifs from the region will require careful excavation of IA sites, which has yet to be undertaken. Ph.D. student and IRAP member, Philbert Katto, will make progress towards achieving this goal.

Stone tools continue to be produced in the Iron Age. They are associated with pottery and slag and appear typologically the same as LSA lithics. The presence of small bladelets and geometric tools produced from a distinctive black chert in Magubike, for example, suggests a preference for higher quality lithic raw material during the Early Iron Age. During this same period, beads made from land snail shells (LSS) appeared in Iringa for the first time. These were made from the shells of giant terrestrial snails (identified as *Burtoa nilotica* in Miller et al. 2018), in much the same manner as OES beads. Four of such giant snail shell beads were excavated from IA levels at Magubike, and yielded direct dates ranging from 1732 ± 23 BP (UOC-4739) to 371 ± 23 BP (UOC-4741) (Miller et al. 2018, p.362). While these LSS beads do date at least to the IA in Iringa, their use may well have continued into the recent period.

### Historic Period

IRAP also documented six sites that had cultural or historical significance, often at the direction of local research participants. This emphasis on consultation and collaboration with the local community has led to the investigation of some of these heritage sites. The majority of the culturally significant sites are associated with the nineteenth-century Hehe armies, and their illustrious leader, Chief Mkwawa (1855–1898). He is known throughout Tanzania for resisting the German colonial entry into the southern highlands of what is now mainland Tanzania (Iliffe 1979, p. 57). Under Mkwawa’s leadership, the Uhehe kingdom became the dominant power in Iringa through a combination of alliances and conquests (Crema 2004). The Mkwawa-led Hehe struggle against German colonial rule was costly for both sides but earned the Hehe much respect for their determination and military prowess (Redmayne 1968).

In addition to a keen interest in Mkwawa, the people of Iringa also have a fascination with footprints. Two footprint sites were brought to our attention. In both cases, they were referenced as being similar to the footprints at Laetoli, the famous ESA site in northern Tanzanian that has attracted the attention of researchers and tourists from around the globe. Although, neither the Tungamalenga site (HxJe-1) nor the Kwilamba site (HxJf-14) turned out to have ancient hominin tracks, we nevertheless documented them as cultural sites. At Tungamalenga, a natural impression in the rock looks vaguely like a footprint, and Kwilamba also has a petroglyph with what seems like a footprint-looking feature (big toe, ball of the foot, and heel strike). Even though these footprint sites are not of archaeological significance, they demonstrate that local people have a keen awareness of the scientific, cultural, and economic value of LSA archaeological heritage.

Many of the heritage sites documented in this article would not be possible without consultation with local communities. While IRAP can survey gullies and rockshelters, there are many important places that are not readily visible, and it is only through collaboration with local people that we can access these places. One example of a concealed site is Mafifi Rockshelter (HxJg-118). It is located near the top of a boulder-strewn mountain and difficult to access. It was said to be a hideout for Mkwawa’s soldiers. The closely spaced boulders create an extensive system of winding passageways that lead up the mountain. We were led to the site through dark passageways for nearly 2 hours before emerging at the top of the mountain where Mafifi Rockshelter is located. The route to the shelter is difficult and dangerous, even with a guide. It would have effectively hidden Mkwawa’s soldiers from German forces, and without the knowledge of the local people, it would also have been hidden from us.

## Highlight of Sites

The 65 new sites reported in this article were recorded over 14 years and seven field seasons, with the participation of numerous graduate students and many dozen local participants. As a result of this collaborative effort, there is considerable variation in the level of detail between site descriptions. In this section, we highlight eleven sites that have good documentation and are noteworthy for their excavation potential or cultural value. We are not reporting on several other well-known sites in this region, including Kihesakilolo/Ikerege and Tavimienda, as they have already been described in detail elsewhere (Itambu 2013, 2015).

### HxJg-125 (Kitwiru Rock Art Site)

**Fig. 7:**
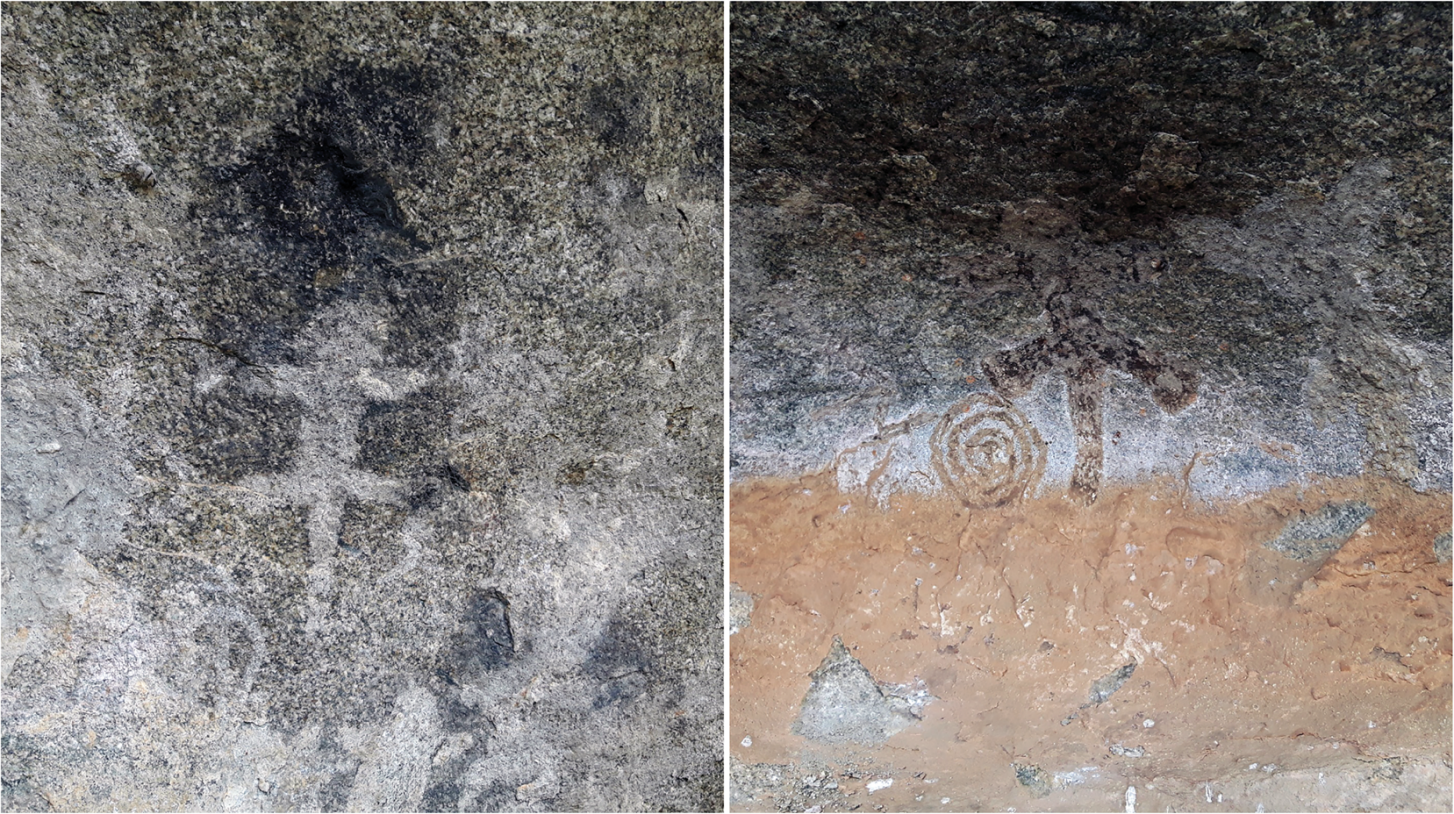
Kitwiru Rock Art panels

The Kitwiru Rock Art site consists of a series of granite boulders on the northern slope of a steep-sided valley. The boulders form several adjoining “rooms” and shelters that contain archaeological materials, including lithics and potsherds. While several artifacts were observed on the surface, the density of finds was very low, and there is evidence of water movement through the shelter. However, the value of Kitwiru is not in its archaeological material but its artwork panel and historical significance. Near the archaeological materials were several images painted on a stone panel, facing south towards the valley and protected from the elements by a rock overhang. Depicted are two silhouettes of what appears to be a lizard, as well as a spiral motif (Fig. 7). There are other unidentifiable stains in association with the art, and it is possible that there are other figures that might be revealed using image manipulation techniques. Several meters from the panel, we discovered a ground stone slab, which may have been used to process pigments or other materials to create the rock art panel.

There is also evidence that the rockshelter has been used recently, and in various ways. For example, a rock-fall trap was set up, presumably to catch small game. We were also informed that the shelter was used in the last several decades by local people to drink pombe (a local home-brewed beer), which was previously prohibited by the colonial government and thus had to be consumed in secret. And finally, the rockshelter is said to have been used as a hiding place by the local people during the 1970s to escape the Ujamaa policy of forced villagization. This refers to the relocation of communities to create centralized villages where services could be better delivered to the people in rural areas. Another rock art site in the same area, Lutona, shows a similar pattern of continuity of use into the present day (Itambu 2013, 2015). Lutona is currently used for iron smelting, and other sites in the area are also used to this day for overnight sleeping accommodations during the rainy seasons (Itambu 2013, p.49).

### HxJh-1 (Ulonge Korongo)

**Fig. 8:**
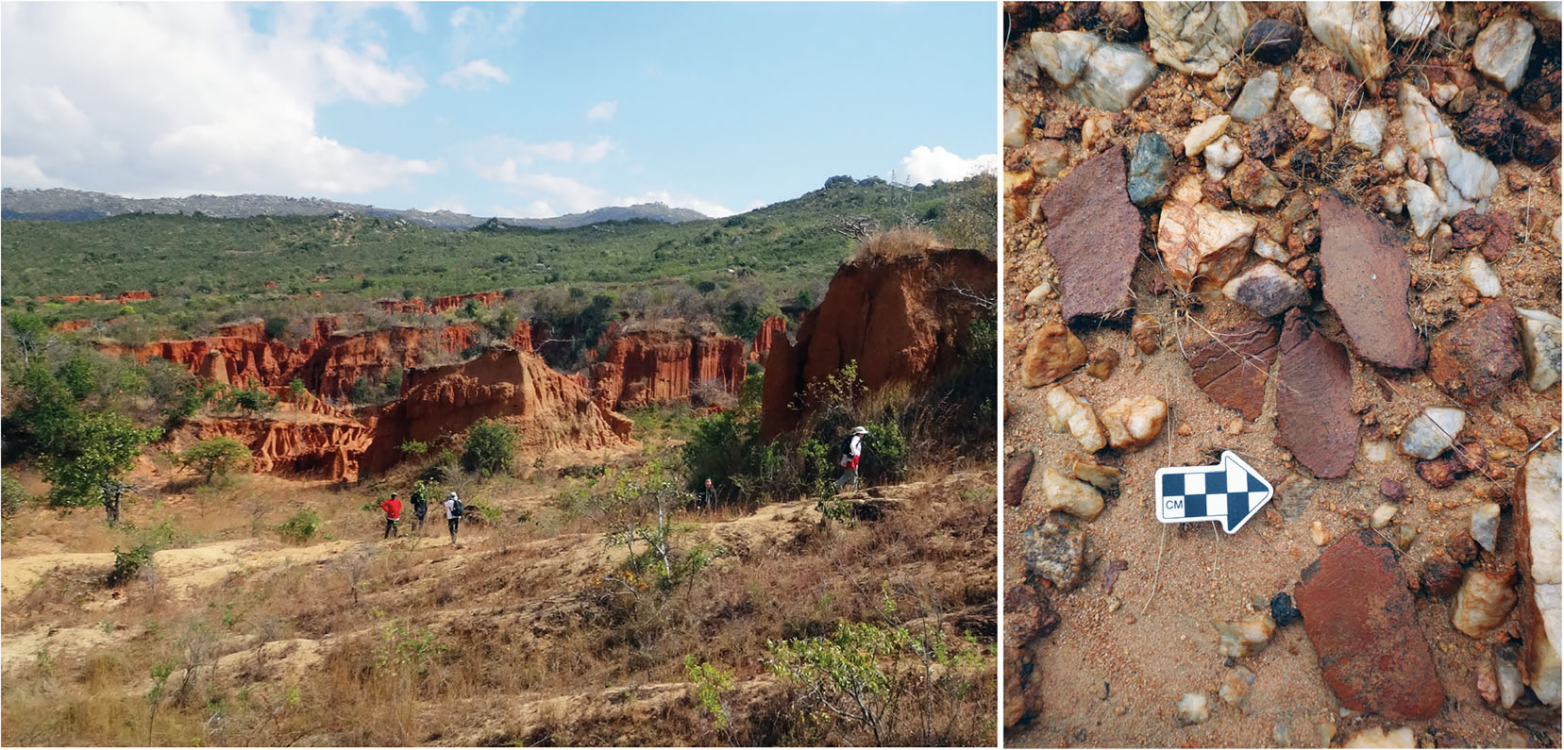
Ulonge Korongo from a distance (left); typical potsherd scatter at Ulonge (right)

HxJh-1 is an erosional gully approximately 140,000 m^2^, located along the base of a hill. The slope is covered with low-quality white quartzite, none of which bears intentional modification, and appears to be a natural outcrop of quartzite rather than the result of human activity. Further downslope, seasonal water movement has cut into the sediments to create a ravine with red pillars and undulating walls (Fig. 8). It is this gully area that contains cultural materials. According to a local belief, Ulonge Korongo is the remains of a king’s palace, and we were informed that it is normally forbidden to take material from the site. Our local guide led us in a ritual to signal our good intentions, thereby allowing us to collect a small sample of potsherds.

The dominant artifact type at Ulonge Korongo is pottery, which suggests a possible historic or IA component, though iron slag is poorly represented. Lithic finds are extremely limited, and the only notable exception is a single quartzite Levallois flake that is denticulated along one edge. The general paucity of lithic evidence suggests that there are no significant ESA, MSA, or LSA components here. However, the abundance of potsherds, particularly large fragments bearing decoration motifs, is compelling. The sherds tend to cluster along the water run-offs and appear to have been washed into the ravine by seasonal water movement. The absence of other contemporary cultural materials (such as iron slag) seems to suggest that the deposits may not be from a habitation site but perhaps represent an intentional pottery cache. Without additional information, it is impossible to say with certainty whether this is an Iron Age, historical, or contemporary accumulation.

Directly adjacent to Ulonge Korongo, we encountered a modern brick-making operation that was mining clay from an exposed edge of the gully. There was also a cramped area where a combination of water movement and digging had made a chamber in the ravine wall. The brick makers called this space Kukifwa Cave and said it was used as a home during the 1974 *Ujamaa* policy of forced villagization. However, aside from a large potsherd, we noted no modern or ancient artifactual scatter.

### HxJh-3 (Matunguu Korongo 1)

Matunguu Korongo 1 is a moderately sized erosional gully, approximately 125,000 m^2^, formed at the confluence of several intermingled streams. This whole area contains an interconnected network of smaller gullies, linked through stream beds to other nearby exposures. During the dry season, the floors of these gullies remain vegetated and even swamp-like because they are fed by small streams. Cattle prints in the mud indicate that this permanent water source is known to local livestock and the people who shepherd them. Despite this year-round presence of water, Matunguu Korongo appears less disturbed by fluvial action than many other gullies we surveyed. The site is farther from nearby mountains than other gullies we surveyed (approximately 500 m), which may reduce the velocity of heavy rainfall coming off the slopes. Several homes and agricultural fields are located near the gully, but it appears otherwise free of human disturbance.

**Fig. 9:**
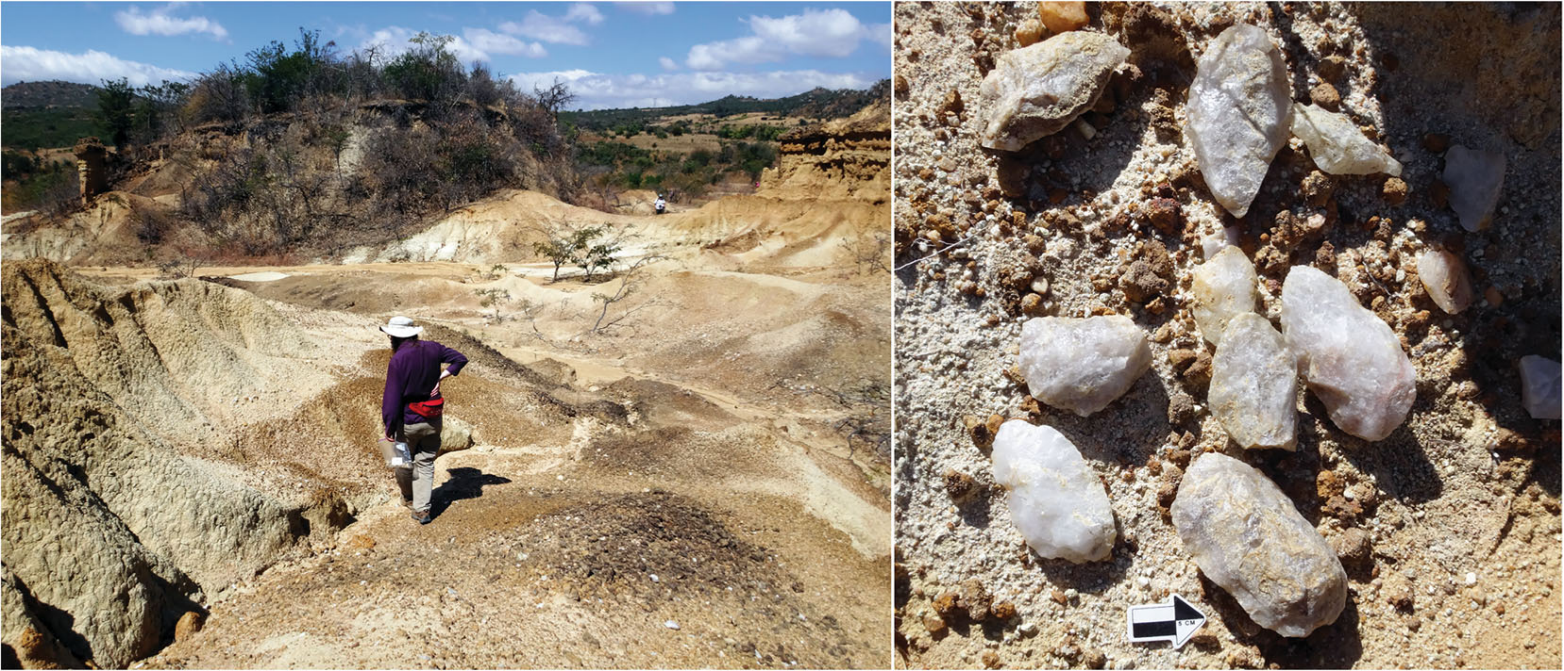
Matunguu Korongo 1 (left); cache of hand axes at Matunguu (right)

This site has some of the highest density of lithic artifacts that IRAP observed in Iringa, outside of the famous Acheulean site of Isimila. The area appears to have been a raw material source, and there are hundreds of cores, primary and secondary flakes with little to no evidence for in situ intensive retouching or shaping. Lithic tools were made exclusively on the locally available white quartzite. Lower quality nodules of quartzite are abundant but seem to have been passed over in favor of more limited, but higher quality, material. Virtually, every piece of superior material is artifactual. ESA and MSA lithics are prevalent, evidenced by diagnostic pieces, including large bifaces, choppers, Levallois flakes/blades, and radial cores. The presence of a small number of decorated potsherds also indicates some later cultural component—Iron Age, historical, or even contemporary. The most notable lithic finds were two small caches of hand axes (*n* = 9 and *n* = 3) nestled among the base of several hoodoos (Fig. 9). Each cache was clustered in a 50-cm^2^ area, and the two groups were only 5–10 m apart from one another, suggesting they may have originally been part of a single collection. The bifaces from the smaller cache are particularly finely shaped in the classic teardrop form, while bifaces from the larger group are more variable and included oblong and ovate shapes, some of which were less refined.

Despite evidence of water erosion and transport, the archaeological potential of Matunguu Korongo is high. Not only is there an incredibly high density of cultural material, but the lag pavement layers we observed may have trapped cultural materials, and protect the underlying deposits. One pavement, in particular, can be traced across much of the profile of the ravine. Through the collection and subsequent refitting of several discrete scatters of lithics, we are confident that many areas have been protected by these lag deposits, leaving many sediments (and the artifacts they contain) relatively undisturbed.

### HxJh-4 (Ilange)

Ilange is an open-air surface scatter, which has been partially exposed by agricultural activity from a bordering sunflower field. Approximately 800 m to the north of the site is the main highway, and 200 m to the south, down a gently sloping valley, is the Little Ruaha River, a year-round source of freshwater. This site was brought to our attention because of its cultural significance as one of the battlefields where Mkwawa fought to consolidate his power as the paramount Chief of the Hehe. While Mkwawa is best known for his resistance against the German colonizing forces at locations such as Lugalo, he also faced internal conflict and resistance to his rule. Oral tradition suggests that at Ilange, Mwambambe Ngondo Kimamula, a subordinate to Mkwawa, fought for control of the Hehe armies. We were also alerted to a significant tree which reportedly marked the medical area for Mkwawa’s injured soldiers. Food and medicines were said to be brought here where the Hehe healer, Mbugi, treated the wounded, and the remains of these supplies can be observed as potsherds around the base of the tree. Oral tradition states that at the conclusion of the battle, Mkwawa beheaded Ngondo Kimamula and several of his generals, and stacked the decapitated skulls in the field.

Local reports suggested the surface at Ilange was still scattered with remains of soldiers from this conflict, and we offered to assist in identifying or documenting the presence of any human material. A section of the site is currently cultivated, and agricultural activity is known to churn the deposits, often exposing underlying archaeological materials. Despite this, we did not note the presence of any human remains or Mkwawa-era material culture on the surface. There were a few fragments of bovid bones on the surface, which looked slightly weathered, and a small (< 3 cm) fragment of a weathered long bone that could not be identified more precisely. Even though there was no material relating to the historic battle observed on the surface, subsurface testing may yet reveal relevant artifacts.

In addition to its historical importance, Ilange also has Iron Age material and possibly LSA and MSA artifacts. A survey of the cultivated field and hill revealed a low to medium density of artifacts over an area of approximately 7000 m^2^. The surface scatter includes the remains of several iron furnaces, potsherds, and various lithic artifacts. Some of the characteristic Iron Age materials, including ceramics, furnace fragments, and iron slag, were concentrated around the edges of some natural granitic outcrops along the periphery of the Ilange hillside. The physical proximity of the potsherds and similarities in their decoration motifs suggest that they may have been complete vessels that were broken in situ.

The Ilange hillside has small amounts of naturally occurring white quartzite, and it appears that the archaeological lithic material is from this local source. All of the lithic artifacts observed were made from a low-medium quality of white quartzite, and some of these have signs of intensive reduction. The quality of the rocks makes it difficult to identify characteristics diagnostic of a particular lithic technology. However, the overall size of the modified products suggests an LSA or MSA component. In particular, Ilange had several radial cores, which are typical of the MSA, but these are much smaller than expected, with some no more than 5 cm in maximum dimension. This diminutive size is comparable with the microlithic industries of the Later Stone Age, but we are not aware of any LSA industries in Tanzania that rely on a radial strategy of blank production. Although these are surface finds and relative ages cannot be inferred, they suggest that Iringa may have a unique industry of MSA micro radial cores. It is also possible that these cores represent the adaptation and persistence of radial core technology into the LSA. Ilange offers a strong prospect for future work, given the combination of the Stone Age, Iron Age, and historical materials in the area. It is possible that the remains of the historic battle and war camp of Mkwawa lie below the surface. Excavation at Ilange could, therefore, provide a unique perspective on the campaigns of Mkwawa against the German forces, and the internal struggles of the Hehe to become a centralized political authority under Mkwawa.

### HxJh-5 (Kihanga Korongo)

Kihanga Korongo is a large (160,000 m^2^) water-eroded gully with an extremely high density of lithic artifacts. It is adjacent to HxJh-3 (Matunguu Korongo), and the two sites are actually joined by a stream that runs through each gully. Some gullies such as Ulonge Korongo have distinct, narrow channels with steeply sided walls and bright red sediments. However, Kihanga Korongo is a broad and flattened exposure with pale tan-colored sediments. This contrast highlights the different depositional and taphonomic circumstances that form gullies in Iringa.

The gully appears to have been a natural source for white quartzite from the ESA onwards. The lithic scatter here is dominated by this local material, with hundreds of cores, flakes, and bifaces, some of which were roughly shaped on-site. There are also low-quality quartzite nodules on the surface, but these are unmodified because the high-quality materials were more selectively exploited. While the sister site (Matunguu Korongo) has lithics made almost exclusively on white quartzite, Kihanga has a wider variety of lithic materials that include some percentage of metamorphic rocks. IRAP collected samples of these less common materials in 2018 to examine whether they are exotic or of local origin.

**Fig. 10:**
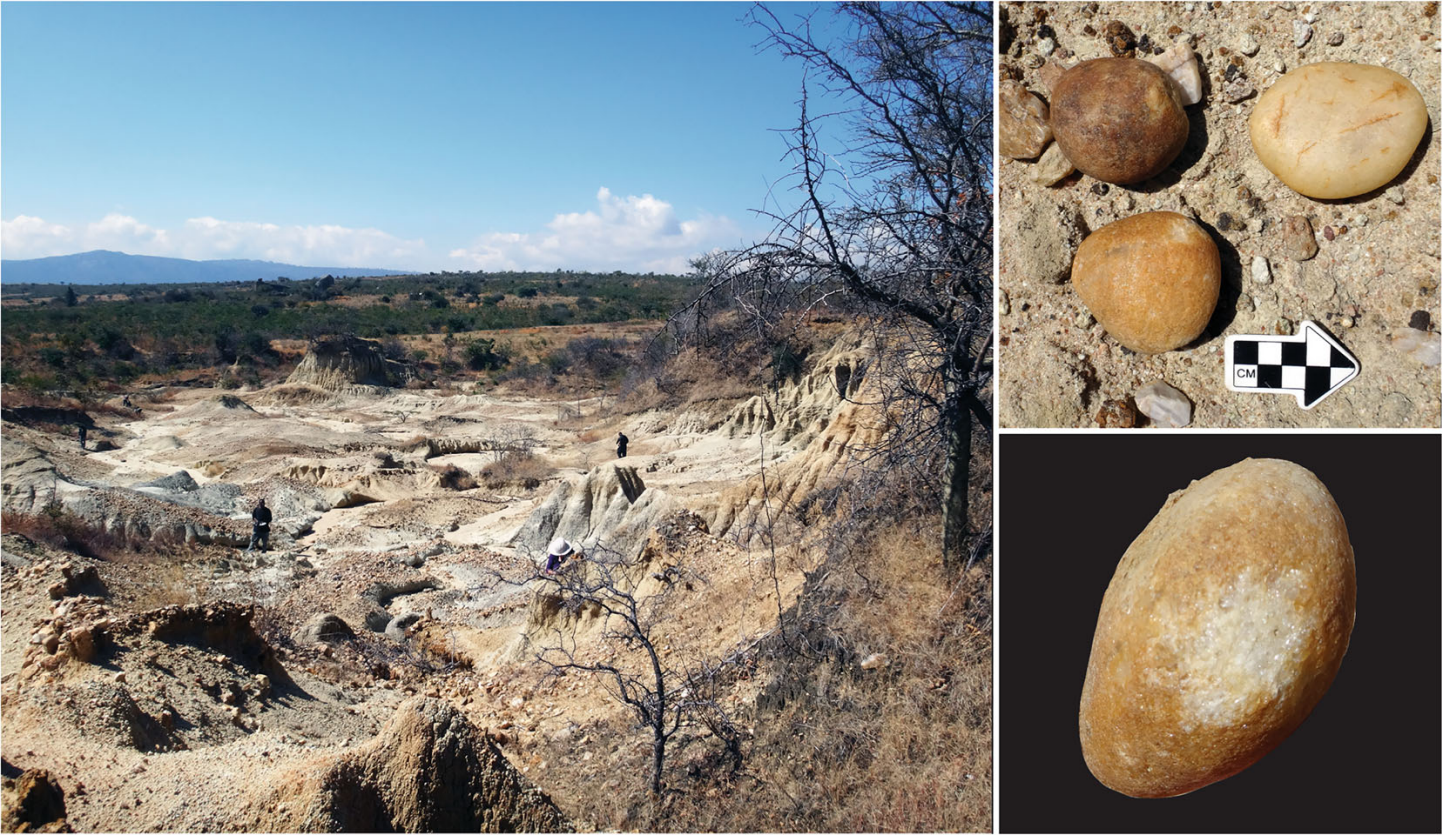
Kihanga Korongo (left); hammerstones from Kihanga (right)

The cultural sequence at Kihanga spans at least 300,000 years. Lithics include diagnostic pieces from the ESA (bifaces and choppers) and MSA (Levallois flakes/points/blades and radial cores). Several scatters of small, LSA-like debitage were observed, but no diagnostic LSA pieces were identified. Hence, the LSA occupation is suspected but not confirmed. A small number of potsherds indicate an IA/historical presence, although several households border the gully, and the pottery could be contemporary. Kihanga is the only place thus far where we have observed clear evidence for hammerstones. One section of the ravine revealed a possible cache, with three water-worn rounded quartzite pebbles showing localized battering (Fig. 10). Despite their presumed prevalence in the Stone Age, hammerstones are surprisingly rare, and these are the best examples we have seen in Iringa.

The excavation potential for Kihanga Korongo is uncertain, though we hope that a more thorough exploration of the gully will reveal suitable sections. The sheer abundance of artifacts at Kihanga and their low degree of weathering suggest that there may be intact stratigraphy nearby. As seen in other gullies, artifact concentrations at Kihanga are strongly associated with lag deposits, which would offer some protection to underlying layers. However, the broad and flat nature of this gully leaves few hoodoos or vertical wall sections to examine. IRAP only surveyed approximately 10% of Kihanga’s area, and we are hopeful that further survey will reveal intact areas that are suitable for excavation.

### HwJh-3 and 4 (Mafinga Rockshelters 1 and 2)

**Fig. 11:**
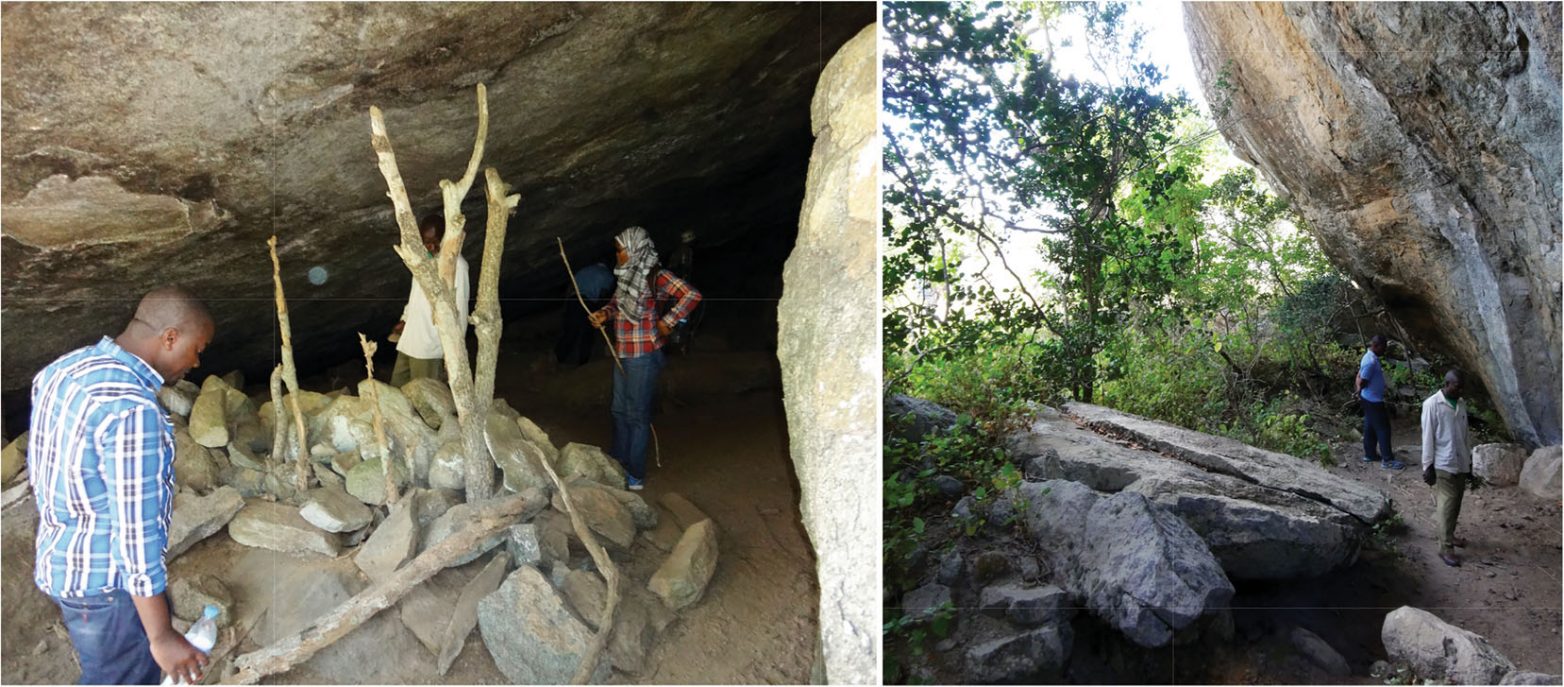
Interior of largest room in Mafinga Rockshelter 1 (left); Mafinga Rockshelter 2 (right)

The Mafinga Rockshelters are located near a mountain chain with multiple granitic outcrops (Fig. 11). The mountain is relatively steep and moderately vegetated with trees. As a result, many of these inselbergs are hidden from view from the ground. The flat area around the mountain is intensively farmed, and herds of livestock can be seen here. There are signs that the mountain is well-traversed, including some footpaths and ax-felled trees.

There is potential for Stone Age material on the mountain. On the lower slopes, low density scatters of white quartzite MSA lithics were observed, including small radial cores and Levallois flakes. This area is steeply sloped, and it appears that the sparse accumulation was deposited by gravity from somewhere higher up the mountain. Closer to the top of the mountain, there is a natural outcropping of low-moderate quality white quartzite, none of which appears artifactual. The source of the MSA lithics was not discovered, but future archaeological survey on the lower- to mid-range of the mountain slope may identify Stone Age sites. The mountain chain is several kilometers long, and satellite images suggest there are many potential overhangs at higher altitudes. However, given the low density of surface scatter, general lack of lithic material, and steep nature of the landform, it may not be worthwhile to explore this area further for the source of the MSA lithics.

Mafinga Rockshelter 1 is a historically significant site, locally known as a place where Mkwawa’s family kept cattle, and occasionally served as a hideout for Hehe soldiers. There are three adjoining “rooms” to this overhang, the largest of which was approximately 4 m wide and 15 m deep and contained what appears to be a barrier roughly constructed with rocks and tree branches. Towards the rear of the shelter, the ground rises to meet the roof, and while the room is too low to walk comfortably, there is still space for people to sit or lie down. There were a few undecorated potsherds and faunal remains (*n* < 10), and nothing on the surface suggested intensive use in the past. Nevertheless, Mafinga 1 is important because of its connection to Mkwawa and the Hehe Rebellion.

Mafinga Rockshelter 2 is a large granite boulder that was encountered by chance as we descended the mountain. The satellite images indicate that the inselberg is approximately 30 m in diameter. The rockshelter is a product of natural weathering that created a 360° overhang like the brim of a sunhat. There is a small amount of surface scatter, most of which points to a historic or Iron Age occupation, including fragments of decorated pottery, animal bones, and a possible grindstone. Some white quartzite is present, but it does not bear obvious signs of modification and may be a natural accumulation. There is low to moderate potential for excavation at Mafinga Rockshelter 2. It is unlikely that the sediments around the rockshelter have sufficient depth to contain Pleistocene deposits, but Iron Age occupations seem plausible.

### HwJg-101 (Kikongoma, Aka “Black Stones”)

Kikongoma, also known as Black Stones (or *Mawe Meusi* in Swahili), is a cultural site along the Little Ruaha River. The site likely gets its nickname from the large metamorphic boulders that are darker than the rock typically found in the region. Local traditions state that Mkwawa’s mother, Sengimba, died by suicide at this location, although there are varying stories about the reason. Some informants believe she killed herself upon hearing that her son had been captured by the Germans, and others suggest she was fleeing from conflict during the Hehe uprising when Mkwawa seized power. The location of her death is supposedly where large boulders form a natural bridge across the river. Despite the gentle movement of water and low levels during our visit, the channel here is deep and probably fills with fast-moving water during the rainy season. Lingering silt has caked onto the boulders and marks the high water level in the past. Piled conspicuously on a nearby rock were grass, leaves, and branches, in varying states of desiccation. These were said to be offerings left to Mkwawa’s mother.

### HwJg-104 (Mlangali Hill)

**Fig. 12:**
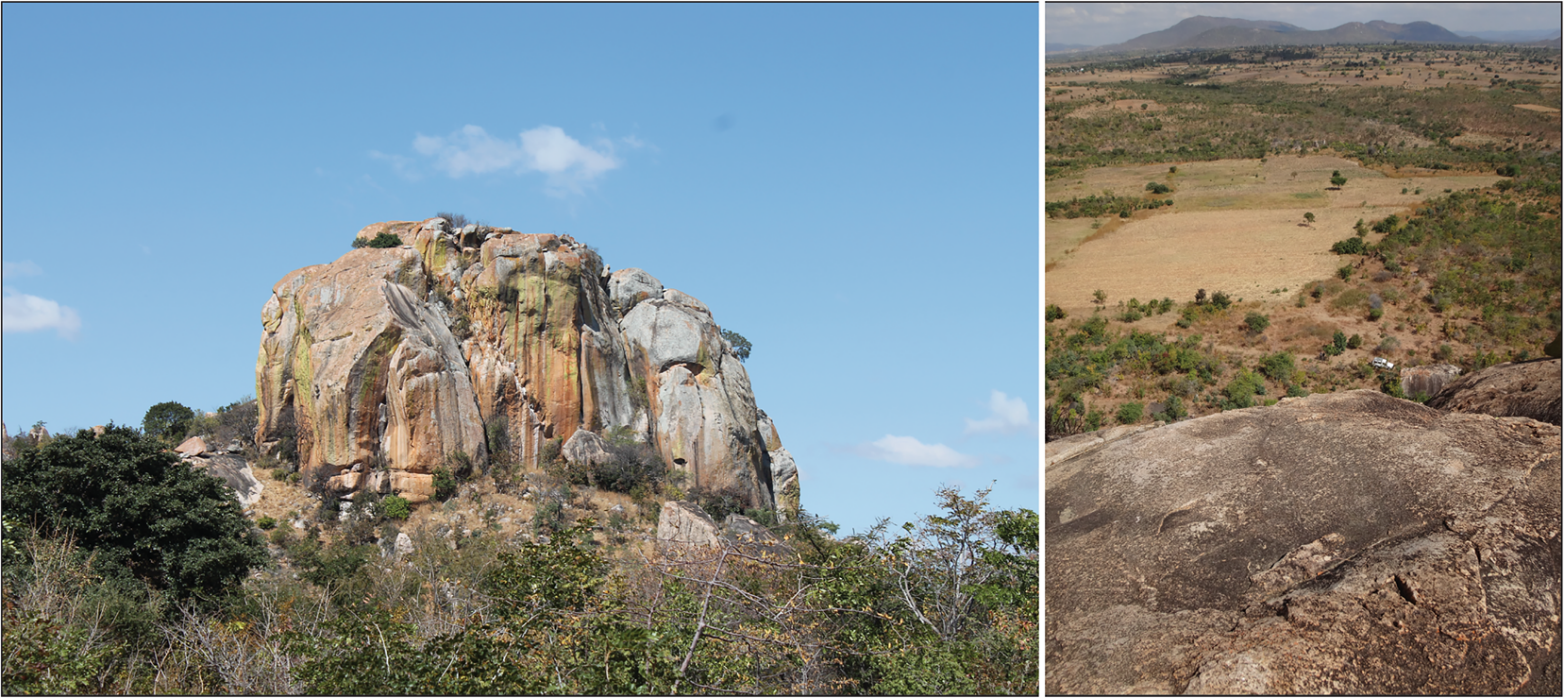
Upper portion of Mlangali Hill (left); looking down from a lower terrace, note the white SUV for scale (right)

Mlangali Hill is a freestanding granitic inselberg of impressive stature (Fig. 12), near the town of the same name. It is only 400 m from the base of another hill, but the surrounding area is otherwise flat, making this outcrop stand out in striking contrast, even from several kilometers away. This landform is particularly eye-catching because the granite seems to rise directly out of the ground. The nearest main road is approximately 2 km from Mlangali Hill; however, much of the surrounding land is cultivated, and informal roadways led us to the base of the inselberg. The surface area of the hill is approximately 37,000 m^2^, much of which is sheer granite with no potential for sediment accumulation. At least two of its approaches have steep but climbable slopes that are moderately to heavily vegetated with trees and shrubs. The top of Mlangali Hill provides a commanding view of the surrounding terrain and has soil development that supports tall grasses, bushes, and even some large trees. Rather than one single flattened area, the hill supports several terraced levels, only a few of which have been explored by IRAP.

Survey around the base of the inselberg revealed a low density scatter of furnace fragments, iron slag, potsherds, and quartzite lithics of an indeterminate industry. No artifact scatter was observed during the short but intense climbs up and down the slope, but the upper reaches of the hill revealed areas with moderate amounts of cultural material. Potsherds, iron slag, burnt bone, and a rusted (modern) bowl were located on the top of the hill, suggesting contemporary and IA occupation. A small number of lithics (*n* < 10) were also observed, which must represent intentional human transport as there is no naturally available raw material source on Mlangali Hill. The lithic assemblage has no diagnostic types, but the presence of a secondary chert flake and two possible Levallois flakes suggests that an MSA component may be located in the area.

There are two types of natural features on Mlangali Hill, which have some potential for excavation: clefts and overhangs. A majority of the large, intact pieces of pottery that we observed were collected in clefts, which formed through the natural weathering of boulders. It is unclear whether the pots were placed in the crevasses, or fell into it from above, but all these clefts contained large fragments of ceramics almost without exception. There is also at least one overhang, with two adjacent rooms, both of which are suitable shelter areas. The first room is approximately 2 m × 2 m and featured a rusted metal bowl and some surface charcoal. No prehistoric artifacts were observed on the surface, but shrubs growing just in front of the entrance suggest the possibility of deep accumulation of archaeological deposits. The second room is about 1.5 m × 3 m and contains several potsherds and two quartzite lithics. Further survey will likely yield additional places on Mlangali Hill with archaeological potential.

### HwJg-105 (Mgera Furnace Site)

The Mgera Furnace Site is an open-air surface scatter, located just a few hundred meters from Mlangali Hill. It is a flat area of red, iron-rich sediment exposed by water run-off from a mountain that borders the site to the east. Most of the surface shows evidence of low-intensity sheet flow, although some deeply incised channels are several meters deep as well. Due to the extensive water movement, Mgera is a palimpsest with IA materials lying directly next to exposed Stone Age lithics. There is also evidence for intensive iron processing. We documented at least half a dozen dense accumulations of iron, iron slag, and furnace fragments, which are likely in situ furnaces, one of which is bisected by the road.

The entire site slopes gently downhill to the west and is more heavily vegetated near the base of the mountain to the east. This mountain features numerous large boulders on its sides that could have served as shelters. We also observed a decline in the proportion of IA materials as we traversed east, and towards the base of the mountain, we found almost exclusively Stone Age lithics. It is unclear whether this pattern represents differences in past land use or taphonomic processes. The excavation potential of the Mgera Furnace Site is uncertain. The density of surface finds and the wide-ranging periods are encouraging, but significant water disturbance is a cause for concern. The IA component of the site appears relatively intact, disturbed mostly by the modern road activity. Shovel testing could reveal whether intact layers below the surface would be worthy of excavation. The deep natural channels could also provide a means of assessing the sedimentology of the site.

### HxJg-107 (Mangalali Hill)

Mangalali Hill is a low mound-like hill, surrounded by cultivated land, located less than 20 m from the main road. The whole site is less than 10,000 m^2^, encompassing the hill and portions of the adjacent sunflower fields, although the full extent has not been explored. The hill has gently sloped sides, with large, flattened areas higher up that would be suitable for occupation, but there are minimal surface finds there. The vast majority of surface finds are located around the periphery of the hill in the agricultural fields, which are highly disturbed by cultivation. Most of the artifacts suggest Iron Age or recent occupations, however there is also some evidence of Stone Age deposits. Even though the site is relatively small, we observed at least six distinct decoration motifs on potsherds on and around the base of the hill. Moving away from the hill, onto a well-worn footpath through the farm fields, are some lithics on good quality material including black chert, orange quartzite, and the typical white quartzite. Large flakes and Levallois technology suggest that MSA deposits may have been churned up by the agricultural activity. Up the slope, two prominent clefts were found to contain pottery sherds, much like the sherds found in the clefts at HwJg-104 (Mlangali Hill). There are also several very small overhangs at Mangalali Hill (none larger than 2 m × 2 m), with surface scatters containing animal bones, lithics, slag, and potsherds. Abundant artifacts litter the surface of the sunflower fields around the base of the hill, indicating that subsurface components once existed, and deeper levels may have intact IA/historical material deposits, and possibly Pleistocene-age deposits. In addition, the small overhangs have some sediment accumulations that could help clarify the stratigraphic sequence of the ceramic decoration motifs in the region.

## Discussion and Conclusion

The Iringa Region has a long and rich history, with at least half a million years of occupation. It was a home for our hominin ancestors, a center of innovation in the MSA, an environmental refugium where people could survive the Last Glacial Maximum, and a stronghold where Chief Mkwawa held off the German colonial army. Today, Iringa is a vibrant and bustling region. It embraces new ideas while maintaining traditional values. Our research in Iringa for more than a decade notwithstanding, there is still much that we do not know about its past.

We have documented ESA, MSA, LSA, IA, and historical materials. Most of our understanding of the cultural sequence focused on the Stone Age, and is based on the lithic typology from two excavated sites. Radiocarbon dating has proven successful in helping us understand some aspects of the settlement history of Iringa, but other dating techniques, including electron spin resonance and optically stimulated luminescence, have failed to yield satisfactory results. Future research should seek to refine the lithic typological sequence by associating it with absolute dates for the various technological periods. Further efforts should also be directed at improving the palaeoenvironmental record for Iringa. These will be especially important for understanding the depositional sequence in gullies, and relating their formation to the artifact-bearing lag deposits.

While our project began as a purely archaeological endeavor, we later included documentation of cultural heritage as a critical part of our research. All of the sites that we identified offer important insights to the past of the Iringa Region, and our goal now is to bring this information to the attention of the wider public, in Iringa in particular and Tanzania more broadly. By working closely with local communities, our project has created a culture of collaboration wherein residents are acknowledged for their roles as knowledge keepers, historians, and producers of their narratives. Public and community-based archaeologies have become increasingly important in Africa because of concerns over loss of cultural heritage in the contexts of colonialism and post-colonialism, development, and conflict (Schmidt and Pikirayi 2016). By sharing our findings not only through academic journals but also directly with the people of Iringa, we hope to support local guardianship of these heritage resources and encourage the development of tourism in the region. Since we began documenting sites and cultural heritage in Iringa in 2006, we have learned much about community outreach. We have adjusted our means and media of engagement to include community posters, pamphlets, and stickers (see Biittner and Willoughby 2012). The next steps include facilitating the development of new museum displays at the Isimila visitor center/museum, and supporting the work of local Antiquities and Cultural Heritage officers financially and with supplies. We also plan to hold sessions with stakeholders throughout the region to discuss our work and share ideas about what we can do to support local initiatives. As our funding comes to an end in 2021, this is a time for reflection by our team. Our project goals must be considered and evaluated in terms of what has been accomplished and what still needs to be done.
